# Forming a Parent And Clinician Team (PACT) in a cohort of healthy children

**DOI:** 10.1186/s40900-021-00293-y

**Published:** 2021-06-27

**Authors:** Shelley M. Vanderhout, Catherine S. Birken, Maria Zaccaria Cho, Jonathon L. Maguire

**Affiliations:** 1 Li Ka Shing Knowledge Institute of St. Michael’s Hospital, Unity Health Toronto, 30 Bond Street, 15CC-014, 209 Victoria St, Toronto, ON M5B 1T8 Canada; 2 Division of Paediatric Medicine and the Paediatric Outcomes Research Team, The Hospital for Sick Children, 555 University Avenue, Toronto, Ontario M5G 1X8 Canada; 3 Child Health Evaluative Sciences, The Hospital for Sick Children, Peter Gilgan Centre for Research & Learning, 686 Bay Street, 11th floor, Toronto, Ontario M5G 0A4 Canada; 4 Patient Partner, Toronto, Canada

**Keywords:** Patient engagement, Partnership, Children, Parents

## Abstract

**Background:**

Engaging parents in child health research can facilitate choosing relevant research questions, recruiting participants who reflect the diversity of large communities, and disseminating study results to communities in accessible ways.

**Main body:**

Primary care well-child visit systems present a foundation for trusting relationships between families and clinicians, lending itself well to a system where health research is embedded into the delivery of health care. We provide an example of a practice-based research network called TARGet Kids!, which is a longitudinal cohort study of children from birth to adolescence. Researchers and clinicians have partnered with parents of children participating in TARGet Kids! to ensure child health research is centred on family values and preferences. A Parent And Clinician Team (PACT) was formed to set research priorities, co-design research protocols, troubleshoot issues, and communicate research to knowledge users.

**Conclusion:**

This partnership will facilitate child health research which is feasible, relevant and inclusive for improving children’s health care and public health policy.

## Plain English Summary

Including parents as partners in child health research can lead to choosing relevant research questions, recruiting children to participate who represent the general population, and sharing study results with communities. The structure of primary care offered to children presents an opportunity for building trusting relationships between families and clinicians, which may encourage parents and health care providers to participate in child health research. We provide an example of an ongoing study called TARGet Kids!, which includes children from birth to adolescence who attend regular health care visits. Researchers and clinicians have partnered with parents of children participating in TARGet Kids! to ensure child health research is centered on family values and preferences. A Parent And Clinician Team (PACT) was formed to set research priorities, design research studies together, troubleshoot issues, and communicate research findings back to health care providers, families, and policy makers. This partnership will lay a foundation for child health research which is practical, relevant to families and inclusive for improving children’s health care and public health policy.

## Background

Involving children in health research is challenging for a multitude of well described reasons, such as hesitancy among parents to consent to participate, strict regulatory requirements for research with vulnerable populations, and limited funding [1]. This often results in low quality evidence with small and un-representative samples, low adherence to interventions and short follow-up duration [2]. The cumulative result is a lack of information to guide children’s health care resulting in considerable practice variation [2]. Engaging parents and caregivers in the co-development of child health research could help overcome these challenges.

Health researchers are encouraged by organisations such as National Institute for Health Research (NIHR) Be Part of Research [3] to include patients and families in the holistic research process. There is growing interest in patient-oriented research and increasing recognition by policymakers of its importance worldwide; for example, the Canadian Institutes of Health (CIHR) Research Strategy for Patient Oriented Research (SPOR) [4] and Patient Centred Outcomes Research Institute (PCORI; USA) [5]. Evidence supports the benefits of patient engagement, including improved patient satisfaction and more accessible and acceptable health care practices [6]. However, challenges can include lack of resources to support organised, sustained patient engagement over long research processes, difficulty reaching representative populations, and perceived tokenism by patient partners [7]. In this article, we describe our experience with developing a partnership with parents who are engaged in a large primary care research network alongside an engaged parent partner.

## Main text

### TARGet kids!

TARGet Kids! [8] is a practice-based primary care research network for children established in 2008, and is a collaboration of researchers, primary care clinicians, health system stakeholders, parents and children. Through TARGet Kids!, longitudinal cohort studies and clinical trials are conducted with the overall aim of keeping children healthy from birth to adolescence, with a focus on nutrition, child development, physical activity, and chronic disease prevention. Clinical trials are embedded in the TARGet Kids! cohort study. Participating children who meet clinical trial eligibility criteria (which varies by trial) are provided opportunities to participate if interested. At select pediatric and family medicine primary care practices in Toronto, Canada which were selected based on interest from primary healthcare providers, families are approached to participate in TARGet Kids! during regularly scheduled well-child visits (up to 15 visits in the first 10 years of life). At each visit, participating families provide a blood sample and information about their growth (height, weight, head and waist circumference), lifestyle (physical activity and sleep habits), development (temperament and behaviour), and nutrition.

### The PACT

The TARGet Kids! Parent And Clinician Team (PACT) was developed in 2018 to engage parents in child health research. The PACT is comprised of 10 parents, a patient engagement facilitator, 1 graduate student, 1 research coordinator, and 5 clinician investigators. The patient engagement facilitator is responsible for maintaining open communication between researchers, clinicians, and parents, supporting parent involvement in research, and contributing to co-building patient engagement into all aspects of the TARGet Kids! research network. Parents are recruited on an ongoing basis through established relationships between researchers and families, “snowball” recruiting between peer parents, and health care provider and research assistant referral. Training was provided to PACT members in the form of online Patient Oriented Research Curriculum in Child Health (PORCCH) modules [9], followed by a group meeting with a guest patient engagement expert. Parents are provided honoraria in the form of gift cards.

PACT members participate in all stages of the research process including identifying priorities, determining research questions, designing study protocols, being co-applicants on all TARGet Kids! funding applications, creating survey instruments and strategies to improve cohort representativeness, informing the feasibility of research in the context of family life, and communicating findings. The PACT meets quarterly, either in person at a hospital or virtually over Zoom, to discuss ongoing study progress, assess readiness and generate ideas for new studies, and review new study protocols. Between meetings, parents communicate with researchers and clinicians over email to provide written feedback on study instruments, protocol design, and messaging about research findings.

Engagement of parents as champions for their children allows TARGet Kids! to focus on research that is most relevant to families. Continual partnership and engagement with the PACT allows clinicians and investigators to understand how parent priorities change with time; i.e., during the COVID-19 pandemic or across different stages of childhood, and with new information; i.e., updated nutrition and physical activity guidelines or education system changes, with the ultimate goal of improving health care delivery. One parent partner described her view of the PACT:

“As parents and advocates for our children, participation in a fantastic initiative such as the TARGet Kids! PACT allows us to feel that we are helping to inform and shape the types of health care services that children receive. It also develops strong partnerships and opens communication between parents and care providers.”

### Identifying priorities

Parents helped to create the TARGet Kids! research agenda through a James Lind Alliance [10] priority setting exercise which identified the top priorities for preventive child health research [11]. Research questions were prioritised by 10 parents and 18 clinicians in a structured workshop. Results showed that compared to clinicians, parents were more likely to be concerned about social media and screen exposure. Parents’ priorities included child nutrition and physical activity, child development, learning, and mental health; clinician priorities were similar but also included access to care and social determinants of health. Each of the identified priorities have guided the development of new studies.

### Designing clinical trials

PACT members are engaged in shaping the design of new clinical trials, including creating minimally burdensome recruitment and consent processes and shaping family-centred interventions for promoting healthy weights in children. Parents have been instrumental in creating verbal study recruitment scripts, shortened consent forms and family-facing study materials. When parents have lived experience related to certain topics, they are encouraged to voice their perspectives and experiences. According to one parent partner:

“*When parents are involved in studies from the beginning of the research process, we perceive a higher degree of credibility in the findings and encourage others to trust health research over less-credible information found elsewhere (online, word of mouth)*.”

### Informing feasibility

TARGet Kids! research takes place during primary care for healthy children, and often relies on data about everyday family routines (for example, nutrition habits, sleep and screen time). It is important that new TARGet Kids! health care interventions and data collection tools are feasible, appropriate and fit into daily life to ensure families feel comfortable participating in research and minimize measurement error. PACT members have informed study feasibility by providing their opinions and suggestions about measurement tools such as questionnaires, smartphone data capture, wearable technologies, and blood spot serology testing for COVID-19. This valuable information, captured early in research planning, also holds potential to improve resource efficiency in research. In one instance, parents indicated they would not be comfortable using a smartphone photo data capture tool for their children, which allowed time and resources to be re-directed to other measurement options.

### Challenges

Institutional buy-in with engaging patients in the research process remains an ongoing challenge. For example, research ethics committees (RECs) tend to be reluctant to take recommendations from patient representatives *as evidence* [4]. Similarly, funders may not provide compensation for the time patient representatives devote to engaging with researchers, which sends mixed messages about the importance of patient engagement in health research. In Canada, the CIHR SPOR is actively advocating for improved communication between researchers and RECs about patient engagement in research, and increased funding opportunities for patient-engaged research [4]. Ensuring that the PACT is representative of the diverse populations that we serve, including ethnic minority groups, households experiencing poverty, and mothers and fathers has also been challenging. While PACT members represent many different neighbourhoods across Toronto, there is room to improve the sociodemographic diversity of the team. To help us strengthen our parent partnership and expand our reach, we are in the process of establishing a diversity framework to champion issues related to equity and inclusion. We are also exploring creative ways to engage fathers, through incentives such as ‘fathers only’ meetings where PACT members who are mothers provide a chance for their partners to attend while they care for children, or alternating mothers and fathers who attend meetings. Engaging children who participate in TARGet Kids! holds possibility for understanding child perspectives about health research. Evidence-based approaches and best practices are not yet available for engaging children as young as those in the TARGet Kids! cohort study, but we look forward to future developments in this area. Disruption to engagement during the COVID-19 pandemic, which caused significant delays in funding decisions, REC review timelines, and changes to recruitment strategies could have been highly challenging. Fortunately, some of these impacts were minimised because of the dedication of PACT members who recognised the increased value and importance of the child health research during this time. The COVID-19 pandemic has also made evaluation of our approach to patient engagement a challenge, but we are in the midst of conducting individual interviews with PACT members to inform improvements in our processes. Finally, it has been challenging to create a compensation policy which fits everyone’s needs and preferences. Development of a parent compensation policy is underway, which will reflect the CIHR guidelines for paying partners in research [12].

## Conclusions

Our experience in developing the PACT indicates that parental input into child health research has been helpful in setting research priorities, designing research protocols, developing data collection instruments, informing feasibility, and communicating research findings to knowledge users. Though we have identified a number of challenges specific to patient engagement in child health research such as diversity, engaging children as research partners, and institutional buy-in, we are hopeful that our work and others’ can contribute to evidence-based approaches to overcoming these. We encourage other child health researchers to build collaborative relationships with families, and support the development of patient engagement policies at research institutions, scientific journals, and funding organisations.

## Data Availability

Not applicable.

## Abbreviations

CIHR: Canadian Institutes of Health Research
NIHR: National Institute for Health Research
PACT: Parent And Clinician Team
PCORI: Patient Centred Outcomes Research Institute
PORCCH: Patient Oriented Research Curriculum in Child Health
REC: Research Ethics Committee
SPOR: Strategy for Patient Oriented Research
